# Eradication of Measurable Residual Disease in AML: A Challenging Clinical Goal

**DOI:** 10.3390/cancers13133170

**Published:** 2021-06-25

**Authors:** Paolo Bernasconi, Oscar Borsani

**Affiliations:** 1 Department of Molecular Medicine, University of Pavia, 27100 Pavia, Italy; oscar.borsani01@universitadipavia.it; 2 Hematology Department, Fondazione IRCCS Policlinico San Matteo, 27100 Pavia, Italy

**Keywords:** AML, MRD, RT-qPCR, MFC

## Abstract

**Simple Summary:**

Relapse is still a major problem in AML because it occurs in about 60–80% of patients, even those who have previously achieved complete remission (CR), defined by the presence of ≤5% bone marrow (BM) leukemic cells. Thus, since CR is unable to predict the relapse risk, significantly more sensitive techniques aimed at identifying AML cells in BM or peripheral blood, a parameter termed measurable residual disease (MRD), have been developed. Among them, RT-qPCR, which analyses appropriate molecular markers, and multiparameter flow cytometry (MFC), which analyses aberrantly expressed antigens, have been identified as the methods of choice for MRD detection. Nowadays, various studies that assessed MRD by these techniques have provided compelling evidence that MRD positivity (MRD+) after standard induction/consolidation chemotherapy and before allo-HSCT is predictive of a very poor clinical outcome. In addition, other studies, which showed that MRD+ clearance even at late time points of the course of the disease may improve the disease clinical outcome, have further strengthened the relevance of MRD+. Thus, a complete MRD eradication, potentially attainable through novel innovative treatments, has emerged as an un-met clinical need in AML and is expected to improve our patients’ prognosis.

**Abstract:**

In non-promyelocytic (non-M3) AML measurable residual disease (MRD) detected by multi-parameter flow cytometry and molecular technologies, which are guided by Consensus-based guidelines and discover very low leukemic cell numbers far below the 5% threshold of morphological assessment, has emerged as the most relevant predictor of clinical outcome. Currently, it is well-established that MRD positivity after standard induction and consolidation chemotherapy, as well as during the period preceding an allogeneic hematopoietic stem cell transplant (allo-HSCT), portends to a significantly inferior relapse-free survival (RFS) and overall survival (OS). In addition, it has become absolutely clear that conversion from an MRD-positive to an MRD-negative state provides a favorable clinical outcome similar to that associated with early MRD negativity. Thus, the complete eradication of MRD, i.e., the clearance of the few leukemic stem cells—which, due to their chemo-radiotherapy resistance, might eventually be responsible of disease recurrence—has become an un-met clinical need in AML. Nowadays, this goal might potentially be achieved thanks to the development of novel innovative treatment strategies, including those targeting driver mutations, apoptosis, methylation patterns and leukemic proteins. The aim of this review is to analyze these strategies and to suggest any potential combination able to induce MRD negativity in the pre- and post-HSCT period.

## 1. Introduction

In non-M3 AML, the cytogenetic/molecular categorization of leukemic cells is the most relevant prognostic parameter [1], and for about forty-five years, the achievement of complete remission (CR), defined by the presence of less than 5% bone marrow (BM) leukemic cells on morphological examination, has remained the primary goal of any AML treatment [2]. However, the cure rate of AML patients in CR is still 30–50% on average, a datum that suggests BM CR is not sufficient to predict the risk of disease relapse [3,4,5,6]. Thus, to improve this gloomy clinical outcome, various techniques aimed at identifying 10^−4^–10^−6^ BM or peripheral blood (PB) leukemic cells (i.e., “Measurable Residual Disease”, MRD) in AML patients in morphological CR have been developed [2,7]. The higher sensitivity of these techniques, which include multi-parameter flow cytometry (MFC), reverse quantitative polymerase chain reaction (RT-qPCR), digital droplet PCR (ddPCR) and Next Generation Sequencing (NGS), has allowed for a deeper definition of remission status, thanks to a multimodal assessment of MRD quantification and the immuno-phenotypic and molecular tracking of leukemic cell defects [5,8,9]. Today, MFC and RT-qPCR (whose features, along with those of conventional cytogenetics, FISH, ddPCR and NGS, are summarized in Table 1) are considered the methods of choice for MRD detection [7], whereas NGS is a relatively new technique with a still low but improvable sensitivity [10]. Beyond their classical assays, MFC and RT-qPCR may identify which patients are at higher risk of relapse by evaluating the percentage of neoplastic cells at diagnosis [11,12,13] and analyzing non-stable molecular markers. These last biomarkers, which flag the genotype, phenotype and function heterogeneity of leukemic initiating cells (LICs) [14,15], may promptly identify unique chemo-resistant AML subpopulations not detected on clinical diagnosis but potentially responsible for disease relapse [16,17,18]. By applying these technologies, various retrospective studies, as well as recent meta-analysis, have proved the negative value of MRD positivity (MRD+) on AML clinical outcome not only in patients submitted to conventional chemotherapy but also in those submitted to allogeneic hematopoietic stem cell transplantation (Allo-HSCT) [19]. In contrast, these same studies and others have revealed that MRD negativity (MRD−) consistently predicts a lower relapse rate (RR) and superior long-term overall survival (OS) [13,20,21,22,23,24,25,26] even if about 20–50% of MRD− patients still relapse. This observation reveals that MRD− as currently assessed does not always provide an absolute certainty of “cure” and strengthens the notion that the MRD threshold should be considered as a moving target that needs to be constantly reset. In addition, MRD assessment is affected by some issues: what is the optimal MRD assay? What is the right time point to perform the assay? What is the influence of MRD status on post-remission treatment strategies? Finally, does the conversion from MRD+ to MRD− during post-remission has any prognostic value? Up until now, this last point has been addressed by one study only, in which post-consolidation MRD− had a significant prognostic influence on five-years relapse-free survival (RFS) and OS independently of the time of its achievement [27]. Despite these limits, MRD is currently employed in the clinic as a routine biomarker for clinical decision making and in clinical trials as a surrogate endpoint to accelerate the discovery and the use of innovative drugs. The development of these innovative drugs, which include small inhibitors of various signal transduction pathways altered by specific gene mutations, regulators of apoptosis, hypo-methylating agents (HMA) and immune checkpoints inhibitors (ICI), remains an area of intensive research in AML. As opposed to standard intensive chemotherapy and allo-HSCT, these new agents allow a more personalized medicine as they specifically target the phenotypic and molecular features of the disease [28]. However, most of these new small molecule inhibitors as single agents have provided unsatisfactory results, probably because multiple “driver” mutations are required for AML development, whereas they have provided successful results when combined with intensive chemotherapy [29]. These findings might suggest that molecular inhibitors, as well as monoclonal antibodies, should be chosen depending on specific mutations or cell surface markers and should always be employed in combination with intensive induction or salvage or maintenance chemotherapy to achieve sounding results. Other investigational drugs that have provided relevant results are those exploring the possibility of overcoming a chemo-resistant phenotype through an immune-based approach and those combining a Bcl-2 inhbitor (venetoclax) with ICI or HMA [5]. This review is aimed at discussing the prognostic MRD power and innovative treatment strategies (excluding cell therapies) which might potentially lead to its clearance. The main studies reporting MRD analysis in AML by molecular markers are presented in Table S1 [21,22,30,31,32,33,34,35,36,37]; those based on MFC analysis of MRD are collected in Table S2 [8,9,13,20,25,27,37,38,39,40,41,42,43,44,45,46,47,48,49,50,51,52,53,54,55,56,57].

## 2. The Prognostic Value of MRD

### 2.1. MRD Assessment after Induction/Consolidation

In contrast to acute lymphoblastic leukemia for which MRD assessment is the standard of care, MRD assessment in AML has always been hampered by difficulties in standardization and uncertainties over its prognostic power [58]. However, various studies have demonstrated that, based on MRD assay results, AML patients in CR can be effectively stratified in different subgroups having distinct RR and OS. The strict link between a positive MRD assay and an inferior clinical outcome has been proved at several time-points during AML treatment history, independently of the MRD assay employed. This tight association has been revealed by past studies [59,60,61] and numerous more recent studies [62,63]. As far as *RUNX1-RUNX1T1* goes, one study employed this molecular marker for disease monitoring at the end of treatment [30] and one during the follow-up period [58]. In one study, BM MRD did not predict subsequent relapse, which was instead predicted by PB MRD [30], whereas in the other study BM and PB MRD were equally effective in predicting relapse [58]. In addition, one study revealed that a >10 copy number reduction of PB *CBFB-MYH11* predicted RR but not OS [31] and noted that a constant increase of MRD during the follow-up period was predictive of morphological disease recurrence. Other studies employed *NPM1* mutation (*NPM1m*) as a molecular marker at different time points [21,22,33,34]. All these studies showed that patients who remained *NPM1* positive (*NPM1*+*)* in PB or BM after the second course of chemotherapy or at the end of treatment presented a higher RR and a lower OS than those of *NPM1*-negative (*NPM1*−) patients. In addition, one study revealed that MRD+ in PB after the second course of chemotherapy was more informative than at any other time point [21]. In patients in CR but still MRD+ in PB after the second course of chemotherapy, multivariate analysis identified *DNMT3A* and *FLT3-ITD* mutations (high-risk mutations) as independent risk factors for RR and OS. Interestingly, MRD+ in PB was associated with a higher Medical Research Council (MRC) risk score and with a higher probability of carrying the *FLT3-ITD* mutation. In addition, in these MRD+ patients, the presence of either the *FLT3-ITD* mutation or the *DNMT3A* mutation determined a clinical outcome significantly inferior than that of MRD− patients, but in patients who were MRD− in PB after the second course of chemotherapy, the presence of either mutation determined a favorable clinical outcome [21]. A validation cohort of ninety-one patients confirmed these results by identifying a subgroup of *NPM1*+ patients without any *FLT3-ITD* mutations who presented a 2-year cumulative incidence of relapse (CIR), significantly higher than that of *NPM1*− patients. This result was also achieved by a French study that evaluated whether post-induction MRD in *NPM1m* patients may identify patients who may benefit from allo-HSCT and fixed the MRD threshold to ≥4 log10 reduction, predicting the lowest CIR and the best OS in multivariate analysis [22]. In this study, MRD analysis performed by searching for *NPM1m* identified a subgroup of *FLT3-ITD*-positive *(FLT3-ITD+)* patients who presented a relatively good clinical outcome, even if, as reported by other studies [64,65], a high *FLT3-ITD* allelic ratio predicted a poor clinical outcome in univariate but not in multivariate analysis. More importantly, in this study, only patients of the not-favorable category who achieved an *NPM1m* PB MRD < 4-log benefited from allo-HSCT. Other studies emphasized the prognostic role of the *FLT3-ITD* allelic ratio, supporting its evaluation, especially from a transplant perspective [65,66].

As already reported, MFC is the other method routinely employed for MRD detection (the most relevant studies performed in pediatric and adult patients that used MFC for MRD detection are listed in Table S2).

The demonstration that MFC-detected MRD+ after induction chemotherapy predicted a high CIR independent of the time at which patients had achieved CR was definitively proven by a study that used a leukemia-associated aberrant immunophenotype (LAIP) approach [13] and by two other studies that employed the same strategy to show the poor prognostic influence of pre-transplant MRD+ [48,67]. In addition, another study revealed that MRD+ identified by MFC through a LAIP approach maintained its prognostic value even at sixteen–eighteen days post-induction chemotherapy [50]. Subsequently, another report that evaluated MRD prior and after allo-HSCT found that only pre-transplant MRD+ was predictive of an inferior RFS and OS, which were not affected by MRD clearance three–five weeks post-transplant [51], an observation that contrasts with a GIMEMA/EORTC study that suggested allo-HSCT might overcome the outcome related to MRD+ [68]. More currently, MFC was employed to determine MRD after the first (C1) and the second course (C2) of intensive induction chemotherapy in *NPM1* wild-type (*NPM1wt*) standard-risk AML to redefine the partial response (PR) and achieve better patient stratification [24]. In this study, MRD status was more predictive for relapse and overall survival at C2 than at C1. After C1, the five-year OS of MRD+ patients in the good and standard risk category in PR was similar and significantly different to that of patients with a resistant disease; after C2, the four-year RFS and the RR of the good and standard risk categories were significantly different between MRD+ and MRD− patients. Importantly, MRD+ patients in these risk categories were the only ones who benefited from allo-HSCT, an observation confirmed by a recent GIMEMA study that used post-consolidation MRD to choose the best post-remission strategy in young de novo AML [56]. This study reported that MRD+ patients in the intermediate risk category were those who benefited from allo-HSCT, which made their disease-free survival (DFS) and OS similar to those of patients in the favorable risk category.

Fewer study used NGS for MRD assessment. A study that performed paired whole genomic or exon sequencing on samples collected at diagnosis and at remission revealed that a variant allele frequency (VAF) ≥ 2.5% identified patients with a poor clinical outcome among those who harbored AML-specific mutations in ≥5% of BM residual leukemic cells [69]. A subsequent study that used the same approach reported that VAF-based mutation clearance (MC) of 1 and a complete MC (CMC) were associated with a significantly better OS and lower CIR and that [70] CMC was associated with significantly better event-free survival (EFS), especially when pre-leukemic mutations were not included in the analysis. The same conclusion was achieved by another study, which suggested that NGS-determined MRD may add prognostic value to MFC-determined MRD [25]. However, since NGS still has a low sensitivity that severely affects its MRD detection threshold, a current study has developed a molecular barcoded approach, which may identify residual mutations at a lower VAF (median VAF 0.33%, range 0.016–4.91%) [10]. This approach was able to precisely identify MRD+ patients who, in multivariate analysis, presented a five-year CIR significantly higher than that of MRD− patients (66% versus 17%). 

### 2.2. Peri-alloHSCT MRD Assessment

As already reported, MRD may be used to guide post-remission strategies, among which allo-HSCT plays a relevant role as post-transplant relapse has an incidence lower than that of post-chemotherapy relapse [19]. Past studies have revealed no difference in the incidence of the post-transplant AML relapse between MRD+ and MRD− patients [71], but peri-transplant MRD status is still considered a critical parameter for post-transplant clinical outcome [72]. Some studies have demonstrated that allo-HSCT can only overcome the poor prognosis of pre-transplant MRD+ in patients included in the low-risk genetic category [26,51,73], whereas other studies have reported that this may also occur for patients included in the intermediate risk category [26,51,73]. The clinical effectiveness of allo-HSCT has been confirmed by a study in which two courses of consolidation or loss of molecular response six months after molecular remission distinguished patients in low- and high-risk depending on *RUNX1-RUNX1T1* MRD status. In this study, high-risk patients who received an allo-HSCT presented a significantly lower CIR and better OS than high-risk patients who did not receive an allo-HSCT [32]. More recently, another study revealed that pre-transplant MRD remained a significant variable for post-transplant outcome in patients with a monosomal karyotype, the statistical influence of which was lost in multivariate analysis [74]. Another study evaluated whether *NPM1* analysis as a pre-transplant molecular MRD could be more effective in predicting a relapse than pre-transplant MFC MRD [37]. After 4.9 years of median follow-up, *NPM1* MRD analysis allowed us to distinguish three patient subgroups with distinct 2-year OS. MRD+ and *FLT3-ITD*+ in the pre-transplant period presented a significantly poor clinical outcome, and by combining these variables, two distinct subgroups of patients with a significantly different two-year OS were identified (17% versus 82%). In addition, a significant reduction in OS was associated with T-cell depletion and MRD+ but was not associated with conditioning intensity and donor source. Even more currently, the prognostic value of CR and MRD status was assessed in relapsed/refractory AML [57]. In this study, CR and MRD− identified patients had the lowest 2-year CIR, better 2-y RFS and a trend toward a better OS. 

Based on these data, many studies have questioned whether conditioning intensity may overcome the poor prognostic significance of MRD+ and influence post-transplant RR. Some retrospective studies have already reported that RR was lower with myelo-ablative conditioning (MAC) than with reduced intensity conditioning (RIC) and with non-myelo-ablative (NMA) conditioning [75,76], whereas other prospective studies have reported that MAC in CR1 patients aged 40–60 years included in the intermediate-/high-risk cytogenetic category did not provide any benefit in comparison with RIC [77,78,79]. Subsequently, it was revealed that in patients who received a MAC regimen after either an umbilical cord blood or a sibling donor HSCT, MRD status did not influence the clinical outcome, whereas in those who received a RIC regimen, MRD status had a significant influence on the RR [80]. This observation was confirmed by a randomized phase 3 BMT CTN 091 study, which enrolled younger patients in morphological CR. This study, which was ceased due to the high RR associated with RIC, established that MAC should be the standard of care for fit patients with AML or myelodysplastic syndrome (MDS) [81]. These results were partially confirmed by a subsequent EBMT retrospective study, which analyzed the clinical outcome of AML patients who received allo-HSCT in CR1 [82]. This study reported that in MRD+ patients aged <50 years, MAC determined an RR lower than RIC/NMA, whereas in MRD− patients aged <50 years, it determined similar clinical outcomes with a higher chronic graft-versus-host disease (cGvHD) incidence. In patients aged >50 years, MAC provided no benefit regardless of MRD status. More recently another study confirmed these results [83]. More currently, and in contrast with the above studies, the Fred Hutchinson Cancer Research Center reported that MAC determines longer RFS and OS but similar RR in comparison to RIC and NMA conditioning [84]. Importantly, in MRD+ patients, MAC determined RR and OS similar to RIC and NMA, whereas in MRD− patients, it determined a better clinical outcome in comparison to RIC and NMA (3-y OS and RFS 69% and 71% vs. 47% and 55% for RIC vs. 47% and 52% for NMA; 3-y RR 18% vs. 30% for RIC and NMA). In addition, in 2020 the EBMT Acute Leukemia Working Party evaluated the curative potential of the conditioning intensity in CR2 AML patients [85]. This study, which performed allo-HSCT from any type of donor, revealed that in patients aged <50 years, MAC and RIC regimens provided similar clinical outcomes, whereas in patients aged >50 years, MAC provided significantly reduced non-relapse mortality (NRM). For completeness, it must be remembered that in relapsed/refractory AML, other studies have explored the role of sequential chemotherapy in addition to RIC in determining MRD clearance but have never achieved this goal [86,87,88].

Another issue analyzed by various studies is whether donor choice may affect allo-HSCT’s ability to overcome MRD+. Among the few retrospective studies that have analyzed this topic, the most relevant one reported that, similarly to MRD+ patients who received the transplant from a matched unrelated donor (MUD), MRD+ patients who received the transplant from a mismatched unrelated donor (mMUD) presented a significantly worse clinical outcome than patients who received a cord blood transplant [89]. In contrast, in MRD− patients, transplantation from either mMUD, MUD or cord blood provided similar clinical outcomes.

### 2.3. How to Achieve MRD Clearance?

#### Consolidation Chemotherapy

EBMT and CIBMTR studies have demonstrated that in patients undergoing MAC or RIC conditioning, pre-transplant consolidation does not provide any advantage as far as CIR and RFS are concerned [90,91,92,93]. However, these studies evaluated neither pre-transplant MRD status nor MRD kinetic clearance, an issue deeply analyzed by GIMEMA/EORTC protocol [9]. This last, which assessed MRD by MFC, underlined the utmost relevance and statistically significant influence of MRD− on clinical outcome independent of the time at which it was achieved and once more underscored the need to maintain MRD−. In this study, consolidation treatment was able to convert MRD+ into MRD− in ten patients who were MRD+ after induction, but it was not able to maintain MRD− in nine patients who were MRD− after induction.

### 2.4. Targeting Driver Mutations and Oncogenic Pathways

#### 2.4.1. FLT3 Inhibitors

Various FLT3 inhibitors distinguished in multi-kinase versus selective FLT3 inhibitors and in type 1 versus type 2 inhibitors have been developed [94]. Monotherapy with type 1 inhibitors, except for sorafenib, has always provided unsatisfactory results. Instead, a study that evaluated sorafenib monotherapy in patients who relapsed post-transplant and in those who relapsed post-chemotherapy reported a complete molecular response of 25% versus 8% and the development of sorafenib resistance in 38% versus 47% of patients, with sorafenib resistance occurring 197 versus 137 days after sorafenib start [95]. Another report suggested that the sorafenib anti-leukemia activity was due to the fact that it induced a high release of IL15 from ITD+ leukemic cells, an event that determined a boost in CD8/CD107a/IFNγ-positive T-cells with a longevity pattern (high Bcl-2 and low PD-1 expression) [96]. In addition, it was reported that three patients who responded to sorafenib treatment administered for a post-transplant relapse presented skin infiltration by CD3+ T-cells and BM infiltration by CD3/CD8/CD279-positive lymphocytes [97].

In comparison to type 1 inhibitors, monotherapy with type 2 inhibitors provided more successful results [98,99]. However, in a phase III study, quizartinib monotherapy did not reach FDA approval, despite the fact that in comparison to salvage chemotherapy it caused a 24% reduction of the risk of death, improved OS (6.2 versus 4.7 months) and the 1-year survival rate (27% versus 20%), as well as allowing more patients to undergo allo-HSCT (32% versus 11%) [100]. Instead, the 37–40% CR rate achieved by gilteritinib monotherapy in phase I/II studies [101] prompted a phase III study that led to gilteritinib approval for relapsed/refractory AML [102]. These successful results and the synergism between FLT3 inhibitors and chemotherapy founded the rational for combining type 2 FLT3 inhibitors with standard induction/consolidation chemotherapy. A phase III study that compared midostaurin plus induction chemotherapy versus chemotherapy alone reported similar CR rates (58.9% versus 53.5%) but superior median OS (74.7 versus 25.2 months), EFS (8.2 versus 3.0 months), DFS (26.7 versus 15.5 months) and 4-year OS (51.4% versus 44.2%) for the midostaurin arm [103]. Midostaurin OS was not affected by the type of mutation whereas EFS was better for TKD+ patients. The results achieved by this study might indicate that midostaurin induces deeper remissions than salvage chemotherapy, a clinical status that should be exploited as soon as possible: patients on midostaurin arm transplanted in CR1 presented a trend towards a better 4-year OS when compared with patients on chemotherapy transplanted in CR1 (63.7% versus 55.7%), an advantage that was lost when patients were transplanted in subsequent CRs. Another phase II study confirmed these results [104]. 

The clinical efficacy of other type 2 inhibitors was revealed by a phase II study (crenolanib as a 1-year maintenance treatment combined with chemotherapy induced a CR in 83% of patients, 80% of whom achieved MRD negativity on MFC) [105] and a phase Ib study (quizartinib) [106]. More currently, a phase I/II study that combined standard induction/consolidation with gilteritinib in two escalating doses fixed the gilteritinib maximum tolerated dose at 120 mg/day and reported a 100% composite CR rate for patients on schedule 1 versus 81.8% for patients on schedule 2 with a median DFS of 297 days [107]. Based on these exciting results, various studies are evaluating the role of these inhibitors as post-transplant maintenance [108]. A study reported that in comparison with historical controls, sorafenib maintenance significantly improved 2-year OS (81% versus 62%), 2-year PFS (82% versus 52%) and 2-year RI (8% versus 38%) and determined a similar 2-years NRM and cGVHD [109]. Another randomized, double-blind placebo-controlled trial confirmed sorafenib efficacy in preventing relapse [110], and a subsequent EBMT retrospective multi-centric study reported that sorafenib maintenance significantly improved 2-year OS (83% versus 62%), 2-year DFS (79% versus 62%), GvHD relapse-free survival (GRFS) and CIR in comparison with historical controls [111]. Based on these data, a randomized, double-blind, placebo-controlled phase III trial is comparing gilteritinib versus placebo [112].

#### 2.4.2. Isocitrate Dehydrogenase 1 and 2 (IDH1/IDH2) Inhibitors

Monotherapy with ivosidenib has been approved for the treatment of newly diagnosed *IDH1+* relapsed/refractory AML patients not eligible for standard chemotherapy due to the results obtained by two studies [113,114]. The first study reported a CR of nine months duration in 22% of patients and a median overall response rate (ORR) of 42%, the duration of which was nine months, as well as no residual *IDH1* mutation in seven patients; the second study reported a CR + CR with incomplete hematological recovery (CRi) of “not reached” duration in 42% of patients and a 64% *IDH1* mutation clearance [114]. Instead, in a dose-escalation and expansion phase I study performed in *IDH2*+ relapsed/refractory AML monotherapy with enasidenib, the oral IDH2 inhibitor determined a 20% CR rate and a 40% ORR [115]. Similar results were also obtained by a subsequent first-in-human study, which reported similar CR and OR rates without any difference between IDH2-R140 and IDH2-R170 mutations, an OS of almost two-years in responding patients and the achievement of transfusion independence. *IDH2* mutation clearance was associated with a 100% clinical response rate and allowed allo-HSCT in 10% of responsive patients [116]. Moreover, in a phase I/II trial, which enrolled older patients with an antecedent hematological disorder, enasidenib induced a 30% ORR and a 18% CR rate [117]. Interestingly when these IDH1 and IDH2 inhibitors were combined with standard chemotherapy in de novo and secondary AML, ivosidenib determined a CR rate of 93% and 73% and enasidenib determined a CR rate of 73% and 63% [118]. More importantly, *IDH1* and *IDH2* mutation clearance occurred in 41% and 30% of patients and MFC-MRD negativity in 89% and 59% of patients.

#### 2.4.3. Inhibitors of the Hedgehog (Hh) Signaling Pathway

Since the aberrant activation of this signaling pathway occurs in many myeloid disorders [119], glasdegib, a potent and selective sonic hedgehog receptor smoothened (SMO) inhibitor which reduces the LIC number has also been tested in AML clinical trials [120]. Various phase I studies have fixed the glasdegib recommended dose to 100 mg [121], and a randomized, open-label, multicenter phase II trial has compared glasdegib plus low-dose cytosine-arabinoside (LDAC) versus LDAC alone, leading to glasdegib + LDAC approval for newly diagnosed AML aged ≥75 years and for patients not fit for intensive chemotherapy due to co-morbidities because of a better clinical outcome with this drug combination [122]. Another phase II trial, which tested glasdegib clinical efficacy in MDS patients who had failed HMA, reported that after a median follow-up of 42.8 months, OR was 6% and median OS was 10.4 months [123], confirmed by another multicenter open-label randomized phase II trial [124]. More recently, a dual center pilot study tested glasdegib’s ability to prevent post-allo-HSCT relapse in AML/MDS who had achieved a stable engraftment twenty-eight days post-transplant [125]. Glasdegib was started after a median time of 46 days post-transplant and was permanently discontinued after a median time of one-hundred forty-two days and continued for one year in eight patients. At day +80, all patients except one did not show any MRD. Relapse was revealed by MFC at a median of 180 days post-transplant, whereas morphological relapse occurred at a median days of 333 days post-transplant. In all patients, 1- and 2-year RFS rates were 41.9% and 31.5% and 1-year CIR (MRD+ and morphological relapses) was 45.2%, whereas in MFC-MRD+ patients, 1- and 2-year RFS were 30% and 16.7% and one-year CIR was 55%. The conclusion of this study is that glasdegib does not reduce the post-transplant relapse probability.

### 2.5. Targeting Apoptotic Pathways

#### 2.5.1. BCL2 Inhibitors

A phase I/II study has revealed that venetoclax, a selective BCL2 inhibitor, combined with HMA or with LDAC yielded 45%/30% MRD negativity in treatment-naïve elderly patients ineligible for intensive chemotherapy who achieved CR/CRi [126,127], a result confirmed by another study [128]. In this study, after a median follow-up of 8.9 months, CR/CRi were 67%, with a CR/Cri of 73% for patients on venetoclax and a median OS of 17.5 months for all patients versus “not reached” for those on 400 mg of venetoclax [128]. The updated results of this study were extremely encouraging: CR/CRi rate was 54%, median OS was 10.1 months, median response duration was 8.1 months and mortality rate was 6% [129]. These sounding results not only led to the approval of these drug combinations in newly diagnosed elderly AML patients but also to the evaluation of the clinical efficacy of venetoclax combined with various schedules of intensive chemotherapy in younger newly diagnosed AML patients. Wei and co-authors [109] reported a CR/CRi rate of 71% (95% in newly diagnosed AML versus 42% in secondary AML) and response rates of 90% for *NPM1*, *RUNX1*, and *IDH1* mutated patients versus 33% for *TP53* mutated patients. At interim analysis, another phase Ib/II study reported a median time of twenty-seven days to achieve the best response and 85% CR/CRi and MRD negativity rates, including a 60% CR/CRi rate in *TP53*-mutated patients [130]. Another phase II study evaluating venetoclax plus two courses with cladribine and LDAC, alternated with two courses of azacitidine, in elderly newly diagnosed AML ineligible for intensive chemotherapy is still ongoing. At interim analysis, CR/CRi and MRD negativity rates were 89% and 84% versus 55% for patients with high-risk cytogenetics [131].

Currently, ongoing studies are exploring venetoclax combined with IDH1/IDH2 and FLT3 inhibitors and with agents restoring p53 activity. A phase I/II study, which explored venetoclax + ivosidenib with or without azacitidine in these AML patients, reported 89% of the overall CR/CRi rate, which varied in relation to venetoclax doses (100% for ivosidenib + venetoclax 800 mg, 67% for ivodenisib + venetoclax 400 mg and 67% for ivodenisib + venetoclax 400 mg + azacitidine) [132]. Since *FLT3-ITD* may determine venetoclax resistance by inducing high BCL-XL and MCL1 levels but in vitro studies have shown a synergistic activity between venetoclax and quizartinib [133], a phase Ib study is evaluating venetoclax + gilteritinib in relapsed/refractory AML [134]. This study reported a preliminary CR/CRi rate of 20% in *FLT3* wild-type patients versus 88% in *FLT3*-mutated patients. Other ongoing studies are evaluating venetoclax + quizartinib (NCT03735875) and venetoclax + HMA + FLT3 TKI in *FLT3*-mutated relapsed/refractory AML (NCT03661307) and in newly diagnosed AML ineligible to intensive chemotherapy (NCT04140487). Venetoclax was also tested in combination with HMA in *TP53*-mutated patients, but results were disappointing [128]. Instead, preclinical studies have suggested an anticancer synergism between venetoclax and idasanutlin, a MDM2 inhibitor [135,136], so that these two drugs were combined in a phase IB study in elderly relapsed/refractory AML patients ineligible for intensive chemotherapy, which provided interesting results [137].

#### 2.5.2. Hypomethylating Agents

Treatment with HMA (azacitidine and decitabine) have been approved for MDS and AML patients ineligible for chemotherapy, and various studies have revealed that HMA could eradicate persistent MRD, especially in high-risk AML patients [138,139]. The RELAZA2 trial, which monitored *NPM1m* and fusion gene rearrangements by RT-qPCR and donor chimerism by flow cytometry on sorted CD34+ cells during a twenty-four-month follow-up period in one hundred and ninety-eight AML/MDS MRD+ patients in CR after chemotherapy or allo-HSCT, has further strengthened azacitidine clinical efficacy [138]. Thirty-one of the fifty-three MRD+ patients who received azacytidine at standard doses for up to twenty-four cycles were relapse-free and alive six month after azacitidine start. In addition, during a median follow-up of twenty-three months, nineteen of the fifty-three MRD+ patients became MRD−, and twelve of these nineteen patients remained MRD− without any clinical relapse. After a median follow-up of thirteen months, RFS at twelve months for all the fifty-three MRD+ patients was 46% versus 88% for MRD− patients. It was noteworthy that a significantly improved RFS and a trend towards an improved OS were noted in patients who converted from MRD+ to MRD−. However, HMA clinical efficacy was not confirmed by other clinical trials [140].

### 2.6. Immune Targets

#### Immune Checkpoint Inhibitors

**Figure 1:**
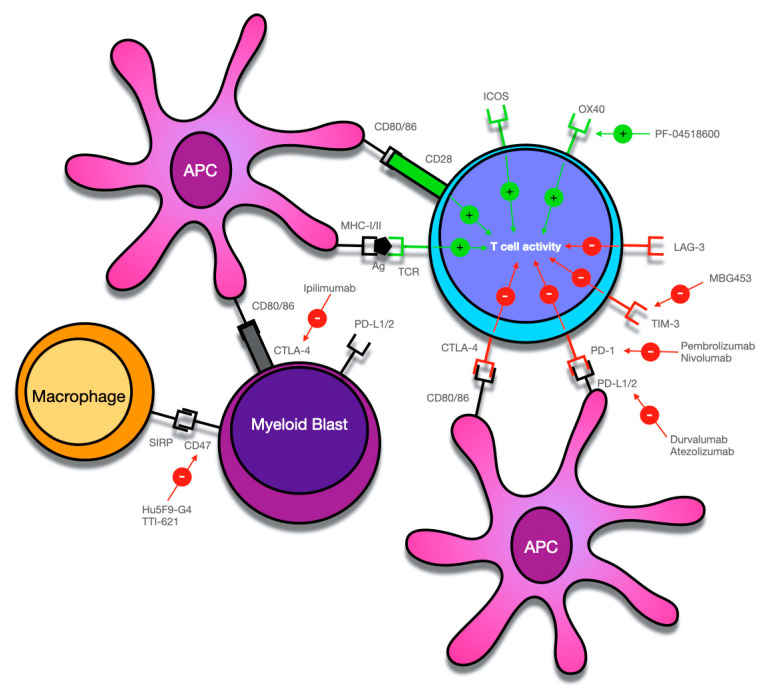
Overview of potential targets for immune checkpoint-mediated therapy in myeloid malignancies. Antigens (Ag), including tumor antigens, are bound to major histocompatibility complex (MHC)-I/II molecules by antigen-presenting cells (APC) and activate T-cells by binding to the T-cell receptor (TCR). Co-stimulatory signals such as activation of CD28 on T-cells by CD80 or CD86 is required for T-cell activation. The activity of T-cells is further regulated by various co-inhibitory (PD-1, CTLA-4, TIM-3, LAG-3) and co-stimulatory receptors (ICOS, OX-40), which can serve as potential targets for monoclonal antibodies that mainly block the activity of co-inhibitory receptors. However, as in the case of PF-04518600, activating co-stimulatory receptors can also be employed to increase the anti-tumor immune response. Myeloid blasts can also express various receptors on their surfaces, which create an immunosuppressive microenvironment and can contribute to immune system evasion. While thus far, T-cells have been the main focus of immune checkpoint-mediated therapy, current research has also identified the interaction of CD47 on APCs and myeloid blasts and SIRPα on macrophages as a potentially targetable mechanism of checkpoint therapy.

Several studies showed that response to ICI required the presence of T-cells within the tumor microenvironment and that the presence and the amount of those T-cells could be a reliable marker to predict the response to ICI in various hematologic and solid malignancies [141,142,143,144]. In line with this evidence, other studies have shown a greater effect of ICI when this therapy was used to treat tumors with a higher expression of PD-1 and CTLA-4 on tumor-infiltrating T-cells and a higher expression of PD-L1 on neoplastic cells. Moreover, data obtained by clinical trials regarding patients with non-small-cell lung cancer and melanoma showed that a significant clinical response could be obtained even in patients with a low expression of PD-L1 [145,146,147]. Furthermore, other studies showed that the co-stimulatory molecules CD80 and CD86 are also expressed by leukemic cells in AML and MDS, but data about the prognostic impact of this finding are controversial [148,149,150] (Figure 1). Subsequent studies demonstrated that those AML patients with a lower likelihood of response to HMA and with a poorer prognosis have a significantly increased expression of PD-L1 and PD-L2 on leukemic cells [151,152]. Increased bone marrow PD-1+ T-cells was found in patients with relapsed AML [153]. Finally, an increased amount of circulating regulatory T-cells in patients with AML and their persistence after induction chemotherapy has been associated with a higher risk of relapse [154,155,156].

In AML, there is a scarcity of data regarding the composition of the tumor microenvironment within bone marrow as far as the expression of immune checkpoint receptors, T-cell functionality, and the distribution of various T-cell subsets are concerned. The mechanism of action of immune checkpoint blockade (ICB) implies the presence of T-cells within the tumor microenvironment. This has been suggested by several studies that reported a significantly increased percentage of T-cells in the bone marrow of AML patients compared with healthy individuals [157,158,159]. Other studies showed that, despite peripheral T-cells seeming to have normal functions in response to co-stimulatory signals, T-cells residing in the bone marrow showed an overexpression of PD-1 (i.e., one of the most important inhibitory immune checkpoint receptor) and use of PD-1 inhibitor could overcome the inhibitory signals transduced by PD-1 [160,161]. 

In AML the differentiation and activation status of T-cells is as important as their number and distribution. The anti-neoplastic activity of CD8+ T-cells is directly based on the release of perforin and granzyme B to induce target cell apoptosis. On the other hand, CD4+ T-cells, depending on the cytokine network in the tumor microenvironment, differentiate into various types of effector cells, even including regulatory T-cells. There are discordant data about the role of CD8+/CD4+ T-cell ratio: in one study, a higher CD8+/CD4+-ratio was found in healthy controls and normalized after chemotherapy, but other studies failed to reproduce this finding [159,162]. One of the mechanisms involved in the immune escape of leukemic cells is called “T-cell exhaustion” and consists of an increased expression of various inhibitory receptors (e.g., TIGIT, LAG-3, TIM-3, PD-1), which hamper normal T-cell proliferation and cytokine production [143]. This mechanism was supported by a recent study, which showed that LILRB4, an inhibitory T-cell receptor, was involved in immune escape by leukemic cells [163]. The mechanism of T-cell exhaustion seems to be prevalent in the bone marrow of patients with relapsed AML and may be a consequence of both persistent antigen stimulation and exposure to multiple lines of prior chemotherapies [160,164,165].

Within the bone marrow, an intricate interplay between myeloid-derived suppressor cells, mesenchymal stromal cells, T-cells and various soluble factors plays a central role in immune regulation [166,167].

Data obtained by preclinical studies suggest that the immune escape of leukemia stem cells may be pursued by activation of the immune checkpoint pathways, and thus treatment strategies based on ICI therapy might be a promising option to eradicate MRD in patients who achieved CR after induction and consolidation therapy [168]. A phase II study (NCT02532231) was conducted in order to investigate the efficacy of nivolumab maintenance in AML patients at a high risk of relapse who achieved CR after induction and consolidation chemotherapy: 1-year OS rate was 86% and grade 3/4 IRAEs was reported in 5 out of 14 patients [169].

Relapse after allo-HSCT is associated with a poor prognosis, and thus treatment strategies aimed at treating and preventing a relapse play a fundamental role in clinical practice [170,171,172]. One of the suggested mechanisms of relapse consists of the downregulation of patient-specific HLA haplotypes, which allows for immune escape from donor T-cells surveillance [173,174]. In a phase I clinical trial (NCT01822509), ipilimumab was tested in patients’ hematologic malignancies who relapsed after allo-HSCT: in this study, 5 out of 28 patients (4 out of 12 AML patients) achieved a CR and with a median 1-year OS rate of 49% [175]. In this study, the safety of ipilimumab in the post-allo-HSCT setting was enlightened by the few cases (only 4 patients) of liver and gastrointestinal GVHD reported [175]. Another clinical trial (NCT00060372) was performed on 29 patients who relapsed after allo-HSCT or after donor lymphocytes infusion (DLI) (of which two patients had AML): this study reported an objective response in only three patients with lymphoid malignancies and in none of the AML patients [176]. Furthermore, a case series reported results obtained by three AML patients who relapsed after allo-HSCT and who were treated with nivolumab: one out of three patients achieved a durable CR, while disease stabilization was reached by another one [177]. 

While the stimulation of the graft-versus-leukemia (GvL) effect by ICB may be beneficial in prevention and treatment of relapse, there are a lot of concerns about the risk of an increased incidence and the severity of GvHD when this therapeutic strategy is adopted in the post-allo-HSCT setting. A fatal GvHD induced by the PD-1 blockade has been repeatedly reported in the post-allo-HSCT setting and seems to have a poor response to therapy with corticosteroids [178,179]. Additional studies to evaluate both the safety and the efficacy of ICI for the treatment of myeloid malignancies relapsed after allo-HSCT are warranted.

## 3. Conclusions

The above reported results obtained by MFC, RT-qPCR and NGS after induction/consolidation chemotherapy and in the peri-transplant period provide convincing evidence that MRD status effectively predicts the risk of relapse and OS/DFS in AML patients, even after allo-HSCT. A progressive substitution of the morphology-based definition of CR with the MRD-based definition of CR will probably allow for an improvement of patients’ clinical outcome. Indeed, MRD-based CR definition will allow us to reach a deeper definition of the remission status. Moreover, continuous development of more sensitive technologies for MRD analysis is required because 50% of patients who are MRD− still relapse [5]. Finally, a wider adoption of an MRD-based definition of CR will allow us to clarify the optimal timing for performing an MRD assay and whether MRD assessment after induction, consolidation and pre-allo-HSCT might have distinct prognostic relevance. More importantly, apart from its prognostic significance, MRD status may function as a guide for clinical hematologists to choose the most appropriate post-remission treatments. Furthermore, use of MRD as a surrogate endpoint for OS will allow for faster development of novel drugs. The second part of this review focused on the most promising targets of MRD-directed treatments. Currently, in AML management, use of BCL2 inhibitors combined with HMA is emerging as one of the most effective strategies for achieving MRD clearance. In *FLT3-ITD*+ patients, the results achieved by combining intensive chemotherapy with FLT3 inhibitors are so exciting that a post-transplant preemptive treatment with these inhibitors has been proposed. The inclusion of high-risk patients in these MRD-directed clinical trials is mandatory to increase our knowledge of how MRD can be employed to drive post-remission treatment strategies and improve our patients’ clinical outcome.

## Figures and Tables

**Table 1:** Technologies for MRD detection in AML.

MRD Assessment Technology	Sensitivity	Advantages	Disadvantages
Conventional Cytogenetics	≈5%	- Standard practice	- Low sensitivity - Time-consuming and labor-intensive - Can be performed only in patients with chrom. abnor. at disease onset (≈50%)
FISH	≈10^−2^	- Useful for numerical chromosomal abnormalities	- Lower sensitivity in comparison to MFC or RT-qPCR - Can be performed only in patients with chrom. abnor. at disease onset (≈50%)
MFC-LAIP	10^−3^–10^−5^	- Sensitive - Extensively available - Can be performed in >90% of patients - Fast	- Need of diagnostic samples - Need of large antibody panels - Does not evaluate immune-phenotypic shifts - Requires technical expertise - Limited standardization across laboratories
MFC-DfN	10^−3^–10^−5^	- Sensitive - No need for diagnostic samples - Results not influenced by immunophenotypic shifts - Can be performed in >90% of patients - Fast	- Requires a significant technical expertise - Limited standardization across laboratories
RT-qPCR	10^−4^–10^−6^	- Sensitive - Well standardized - Common practice in various laboratories	- Suitable targets in <50–60% of patients (in <35% of elderly patients) - Many mutations not suitable for MRD (e.g., FLT3) - Time-consuming and labor-intensive - In some cases, results after several days
ddPCR	10^−4^–10^−6^	- No need for a standard curve - Simultaneous amplification of multiple markers - Higher sensitivity and specificity than RT-qPCR - Easier interpretation of the results - Fewer problems with potential inhibitors of RT-qPCR performance	- Suitable targets in <50–60% of patients (in <35% of elderly patients) - Many mutations not suitable for MRD (e.g., FLT3) - Less time-consuming and labor-intensive than RT-qPCR - Limited numbers of experienced laboratories - Not recommended outside clinical trials
NGS	Extremely variable (1–10^−6^)	- Sensitivity potentially high and dependent on the technology applied - Can simultaneously examine multiple genes	- Low sensitivity with common platforms - Persistence of pre-leukemic mutations may be confounding (e.g., CHIP) - In some cases, results after several days - Expensive - Not standardized - Bioinformatics required for interpreting the results

DfN, different from normal; LAIP, leukaemia-associated immunophenotype.

## Supplementary Materials

The following are available online at https://www.mdpi.com/article/10.3390/cancers13133170/s1, Table S1: Most relevant MRD studies in AML with molecular markers, Table S2: most relevant MRD studies in AML with MFC.

## Abbreviations

allo-HSCT	Allogeneic hematopoietic stem cell transplantation
BM	bone marrow
cGvHD	chronic graft-versus-host disease
CMC	complete mutation clearance
CR	complete remission
CRi	complete response with incomplete hematological recovery
CIR	cumulative incidence of relapse
DfN	different from normal
ddPCR	digital droplet PCR
DFS	disease-free survival
DLI	donor lymphocytes infusion
EFS	event-free survival
GvL	graft versus leukemia
GRFS	GvHD relapse-free survival
Hh	Hedgehog
HMA	hypomethylating agents
ICB	immune checkpoint blockade
ICI	immune checkpoint inhibitors
IDH1/2	Isocitrate dehydrogenase 1/2
LAIP	leukemia-associated aberrant immunophenotypes
LIC	leukemic initiating cells
LDAC	low-dose Cytosine-Arabinoside
MUD	matched unrelated donors
MRD	measurable residual disease
mMUD	mismatched unrelated donors
MFC	multi-parameter flow cytometry
MC	mutation clearance
MAC	myelo-ablative conditioning
MDS	myelodisplastic syndrome
NGS	Next Generation Sequencing
NMA	non-myelo-ablative conditioning
NRM	non-relapse mortality
NPM1m	NPM1 mutated
NPM1wt	NPM1 wild-type
ORR	overall-response rate
OS	overall survival
PR	partial response
PB	peripheral blood
RIC	reduced intensity conditioning
RR	relapse rate
RFS	relapse-free survival
RT-qPCR	reverse quantitative polymerase chain reaction
SMO	sonic hedgehog receptor smoothened
VAF	variant allele frequencies
