# Supplementary material for: Eradication of Measurable Residual Disease in AML: A Challenging Clinical Goal
Source: Cancers (Basel). 2021 Jun 25;13(13):3170. doi: 10.3390/cancers13133170 (PMC8268140; doi:10.3390/cancers13133170)
Supplement: Supplementary file 1 [file cancers-13-03170-s001.zip › cancers-1270621-SI.pdf]

Supplementary Table S1. Most relevant MRD studies in AML with molecular markers.

Molecular marker	Patients, total	Time point	PB/BM	Favorable cutoff (percentage of patients)	Linked risk	Determination limit	Reference
RUNX1-RUNX1-T1	94		PB	Negative (70%)	4-y CIR 23.6% (vs 50.9% if positive), 4-y OS 96% (vs 63.6% if positive)	10^{-5}	28
RUNX1-RUNX1-T1	94		BM	Negative (30%)	4-y CIR 28.2% (vs 32.8% if positive), 4-y OS 86.4% (vs 87.7% if positive, n.s.)	10^{-5}	28
RUNX1-RUNX1-T1	163		PB	<100 copies/10^5 ABL copies (85%)	5-y CIR 7% (vs 100% if ≥100), 5-y OS 95% (vs 59% if ≥100)	10^{-5}	29
RUNX1-RUNX1-T1	163		BM	<500copies/10^5 ABL copies (83.5%)	5-y CIR 7% (vs 100% if ≥500), 5-y OS 94% (vs 57% if ≥500)	10^{-5}	29
RUNX1-RUNX1-T1	137	After 2nd consol. or loss of molecular response after six months of MMR	BM	<0.4% RUNX1/RUNX1T1 transcripts	5-y CIR 5.3% (vs 22% if ≥0.4%), 5-y DFS 94.7% (vs 61.7% if ≥0,4%, 5-y OS 100% (vs 71.6% if ≥0.4%)	10^{-5}	30
CBFB-MYH11	115		PB	<10 copies/10^5 ABL copies (80%)	5-y CIR 36% (vs 78% if ≥10)	10^{-5}	20
CBFB-MYH11	115		PB	<10 copies/10^5 ABL copies (69%)	5-y CIR 7% (vs 97% if ≥10), 5-y OS 91% (vs 57% if ≥10)	10^{-5}	29
CBFB-MYH11	115		BM	<50 copies/10^5 ABL copies (73%)	5-y CIR 10% (vs 100% if ≥50), 5-y OS 100% (vs 25% if ≥50)	10^{-5}	29
NPM1	194		PB	Negative (84%)	3-y CIR 30% (vs 82% if positive), 3-y OS 75% (vs 24% if positive)	10^{-5} (range $10^{-3.7}$-$10^{-7.1}$)	21
NPM1	137		BM	Negative (19%)	4-y CIR 6.4% (vs 53% if positive), 4-y OS 90% (vs 56% if positive)	10^{-5}-10^{-6}	31
NPM1	82		BM	Negative (26%)	3-y OS 84% (vs 76% if NPM1/ABL ≤1% vs 47% if NPM1/ABL >1%)		32
NPM1	194		PB	Negative (92%)	3-y OS 80% (vs not evaluable if positive)	10^{-5} (range $10^{-3.7}$-$10^{-7.1}$)	21
NPM1	131		PB	≥4 log10 reduction (55%)	3-y CIR 20.5% (vs 65.8% if <4log10 reduction), 3-y OS 91-93% (vs 40.8% if <4log reduction)	0.01%	22
NPM1	129		BM	Negative (48%)	4-y CIR 15.7% (vs 66.5% if positive), 4-y OS 80% (vs 44% if positive)	10^{-5}-10^{-6}	31
NPM1	80		BM	Negative (49%)	1-y CIR 37% (vs 63% if NPM1/ABL ≤1% vs 85% if NPM1/ABL >1%), 2-y OS 82% (vs 61% if NPM1/ABL ≤1% vs 45% if NPM1/ABL >1%)	10^{-5}	32
NPM1	136		BM	<200 copies (68% of pts have completed treatment)	No relapse	10^{-5}-10^{-6}	31
NPM1	107	After 4.9-y of median follow-up	PB and BM	≤1 log10<br>In PB: <200 copies/10^5 ABL copies<br>In BM: <1000 copies/10^5 ABL copies	2-y OS 83% (vs 45% if ≥1 log10 in two consecutive samples)<br>PB: 2-y OS 81% (vs 54% if 0.1-200 copies/10^5 ABL copies vs 12% if >200 copies/10^5 ABL copies)<br>BM: 2-y OS 84% (vs 56% if 0.1-1000 copies/10^5 ABL copies vs 22% if >1000 copies/10^5 ABL copies)<br>After combining PB with BM: 2-y OS 83% (vs 63% vs 13)	10^{-5}	35
WT1	129		PB or BM	≥2 log reduction (62%)	5-y CIR 40% (vs 75% if <2log)	10^{-4}	33
WT1	584		BM	<10 copies (32%)	3-y CIR 25% (vs 45% if 10-100 copies vs 72% if >100 copies), 3-y OS 72% (vs 59% if 10-100 copies vs 30% if >100 copies)	10^{-4}	34

Supplementary Table S2: most relevant MRD studies in AML with MFC.

Ref.	Multicenter study, y/n	Demography A/C	LAIP, %	Pts total	MRD eval.	MRD cutoff			Stat. significant variab. on univariate analysis	Stat. significant variab. on multivariate analysis	Study details
						Induction	Consolidation	Post-HSCT			
8	N	C	46	53	I, C	<0.05%	0.2%		RFS, OS	RFS	
36	Y	A	70	56	I, C	0.045%	0.035%		I-C: RFS, OS	I-C: RFS, OS	
37		A	75	126	I	<0.01%<br>0.01%-0.1%<br>0.1%-1%<br>>15	-<br>-<br>-<br>-		RFS, OS	RFS	MRD >1% : 3y RR: 85%<br>MRD 0.1%-1.0%: 3y RR: 45%<br>MRD 0.01%-0.1%: 3yRR:14%<br>MRD <0.01%: 3y RR: 0%
38	Y	C	?	252	I1	0.5%			RFS, OS	RFS, OS	3y OS 69% (MRD neg) vs 41% (MRD pos)
39	Y	A	100	106	Day 16	Log diff. 2.11			CR, EFS, RFS, OS	EFS, OS	
40	N	A	100	62	I, C	Log diff. 2.11	Log diff. 2.53		I: RFS<br>C: RFS, OS	I: RFS<br>C: RFS	
9	Y	A	89	100	I, C	0.035%	0.035%		I,C: RR, RFS, OS	I, C: RR, RFS, OS	5y RFS 72% (MRD neg) vs 11% (MRD pos)
41	Y	A, C	?	150	Day 15, I, I2, C	0.1%-0.2%	0.1%-1.3%		Day 15, I: RFS	_	MRD similar EFS due to traditional risk factors
27	Y	A	?	142	I, C	0.035%	0.035%		I , C: RFS, OS	I, C: RFS, OS	5y RR 60% (MRD pos) vs 16% (MRD neg)
42	N	A	94	54	I, C	0.15%	0.15%		I : RFS, OS<br>C : RFS , OS	I : RFS, OS<br>C :-	
43	Y	C	?	94	I1, I2, C, at the end of treat.	<0.1%<br>0.1%-0.5%<br>>0.5%			I: RFS, OS	I1: RFS, OS	3y RFS 64% (MRD pos) vs 14% (MRD neg)
44	Y	C	100	188	I1, I2, at the end of treat.	>0%, 0-1%			I1: OS, RFS<br>I2: OS, RFS	I1: OS, RFS<br>I2: RFS, RR	3y RR 60% vs 29%
45	Y	C	?	203	I1, I2, at the end of treat.		<0.1%<br>0.1%-1%<br>>1%		I1: EFS, RFS<br>I2: EFS, RFS	I1: EFS, RFS<br>I2: EFS, RFS	MFC is superior to morphology for MRD evaluation
13	Y	A	89	517	I1, I2	<0.1%	<0.1%		I1: RFS, OS<br>I2: RFS, OS	I1: RFS, OS<br>I2: RFS, OS	All the cutoffs between 0.05% and 0.8% are significant
20	Y	A	93	427	I1, I2	<0.1%	<0.1%			I1: RFS, OS<br>I2: RFS, OS	3y OS 38% (MRD pos) vs 19% (MRD neg) after cycle 2
46	N	A, C	100	253	Pre-HSCT	<0.1%			DFS, OS		MRD predictive in CR1 and CR2
47	Y	A	?	210	I, C	0.035%	0.035%		I, C: DFS, OS	I, C: DFS, OS	MRD neg is associated with 5y DFS 57% vs 135 in elderly AML
48	N	A	31	178	Day 16-18 of I	0.15%			RFS	RFS	5y RFS 43%(MRD neg) vs 16% (MRD pos)
49	N	A	?	279	Pre-allo-HSCT and post-allo-HSCT (day +28)			Any measurable MRD considered positive	Pre-HSCT: RFS, OS<br>Post-HSCT: RFS, OS	Pre-HSCT: RFS, OS<br>Post-HSCT: RFS, OS	MRDpos pre-allo-HSCT and neg post : 3y OS 29% and RFS 18%<br>MRD pos at both time points: 3y OS 19% and RFS 14%<br>MRD neg at both time points: 3y OS 76% and RFS 71%

50	N	A	?	359	Pre-HSCT and outcomes post-allo-HSCT	0.1%				OS, PFS, RFS	3y RR 67% (MRD pos) vs 22% (MRD neg)
51	Y	C	78	101	Day 15, pre-C	0.1%	0.1%		Day 15: EFS, OS<br>Pre-C: EFS, OS	Day 15: EFS, OS<br>Pre-C: EFS, OS	5y EFS 65% (MRD neg) vs 22% (MRD pos)
52	Y	C (1-21)	?	216	I1, I2	<0.1%<br>0.1%-1%<br>>1%	-<br>-		EFS<br>OS	I1, I2: EFS<br>I1, I2: OS	I1: 3y CIR 38.6% (MRD pos) vs 16.9% (MRD neg)<br>I2: 56.3% vs 16.7%
25	Y	A	MFC-LAIP plus NGS	430	I1	0.1%			RR, RFS, OS	RR, RFS, OS	Both pos. 4y RR 73%<br>NGS pos/MFC neg: 4y RR 52.3%<br>NGS neg/MFCpos: 4y RR 49.8%<br>Both neg : 4y RR 26.7%
53	N	A	MFC-LAIP plus LSC	242	I2	0.1%<br>LSCs defined as CD34+/CD38-		<0.1%	OS, CIR	OS, CIR	Both neg: 3y OS 66%, CIR 35%<br>MRD pos/LSC neg: OS 68%, CIR 43%<br>MRD neg/ LSC pos: OS 53%, CIR 53%<br>Both pos: OS 0%, CIR 10%
54	Y	A	MFC-LAIP plus RT qPCR	371	C	8 Color MFC assay			OS, DFS	OS, DFS	Both neg: 2y OS 89%, DFS 69%<br>MFCpos/PCRneg or MFCneg/PCRpos: 2y OS 88-89%, DFS 65-76%<br>Both pos: 2y OS 55%, DFS 22%
55	N	A	100	606 (11% with MK)	Pre and post-allo-HSCT		<0.1%	<0.1%	For MRD- pts 3-y CIR, 3-y RFS, 3-y OS, 3-y CI of NRM 22%, 58%, 62%, 20% (vs 66%, 18%, 32%, 16% for MRD+)<br>for MRD- MK pts 3-y CIR, 3-y RFS, 3-y OS, 3-y CI of NRM 46%, 46%, 43%, 13% (vs 72%, 9%, 15%, 19% for MRD+ MK pts)<br>for MRD- non MK pts 3-y CIR, 3-y RFS, 3-y OS, 3-y CI of NRM 20%, 60%, 64%, 20% (vs 64%, 21%, 38%, 15% for MRD+ MK pts)	Pre-HCT MRD+ vs pre-HSCT MRD-, remission status (I CR vs IICR), conditioning regimen (RIC/NMA vs MAC) significant for relapse, failure for RFS, overall mortality; MK (yes vs no) n.s.	Significant difference between MK+ and MK- for adverse risk and complex cytogenetics, WBC count at diagnosis, secondary AML, duration of CR, percentage of MRD+ pts
35	N	A	100	141	After salvage therapy and post-allo-HSCT		<0.1%	0.1%	CR pts with lower CIR, better RFS and similar OS in comparison to CRi/MLFS pts<br>Similar trend for MRD- pts; the level of MRD- did not affect CIR, RFS and OS<br>HSCT strongest factor for CIR, RFS and OS; HSCT higher in pts who achieved CR vs CRi/MLFS and MRD- vs MRD+<br>Pre-transplant MRD status not associated with differential outcomes in relation to CIR, RFS and OS	Hematological recovery and MRD allow to distinguish three pts category with different 1-y CIR (47% vs 67% vs 76%) and median RFS (10.1 vs 5.1 vs 4.2 months)<br>CR MRD- associated with significantly lower 2-y CIR (58% vs 73%), better 2-y RFS (30% vs 15%) and a trend towards better 2-y OS (37% vs 21%)	No different rates of MRD- patients who achieved CR, CRi and MLFS
